# COVID-19 in Baghdad, Iraq: adaptive and emotional findings in a household cluster survey

**DOI:** 10.3389/fpubh.2023.1130227

**Published:** 2023-11-30

**Authors:** Riyadh Lafta, Sahar Al-Shatari, Meighan Mary, Gilbert Burnham

**Affiliations:** ^1^College of Medicine, Al Munstansiriya University, Baghdad, Iraq; ^2^Human Development and Training Center, Ministry of Health, Baghdad, Iraq; ^3^The Johns Hopkins Bloomberg School of Public Health, Baltimore, MD, United States

**Keywords:** COVID-19, Iraq, COVID-19 household impact, COVID-19 economic impact, COVID-19 psychological impact

## Abstract

**Purpose:**

The objective of this study was to assess the impact of COVID-19 infection on households in Baghdad, Iraq.

**Methods:**

A cross-sectional household survey was conducted in early 2022; 41 clusters were selected proportional to population size from the districts of the Baghdad governorate. Households were randomly selected for inclusion. The head of household or senior female member present was interviewed to obtain a listing of COVID-19 infections, deaths, and vaccinations among members of the household and to understand if social and economic changes occurred during the pandemic. All analyses incorporated the complex survey design and sample weights for clustering.

**Findings:**

The findings revealed that there were 1,464 cases of COVID-19 (37.1%) and 34 reported fatalities among the 927 households enrolled in this study. One or more COVID-19 immunizations were received by 50.9% of household members. Preventive measures against COVID-19 were widely reported to be being practiced but were not more commonly reported in households having reported a clinical case of infection. While some households where infections had occurred stated that their household expenses were increased, overall, infections were not associated with significantly increased household costs. In households where COVID-19 had occurred, senior members reported a substantial increase in emotional and psychological problems compared with uninfected households.

**Implications:**

COVID-19 deaths were rare, though infections were common, suggesting an effect of vaccination and other efforts. The household economic implications were minimal in houses with and without COVID-19-infected members. COVID-19 had mental health consequences on affected and unaffected populations alike. It is conceivable that the fear and uncertainty generated by the pandemic had an effect on senior household members which was out of keeping with the other effects in the households sampled. This suggests that there may be a persisting need for mental health services for a protracted period to manage the consequences of mental health needs arising from the pandemic.

## Introduction

**Figure 1:**
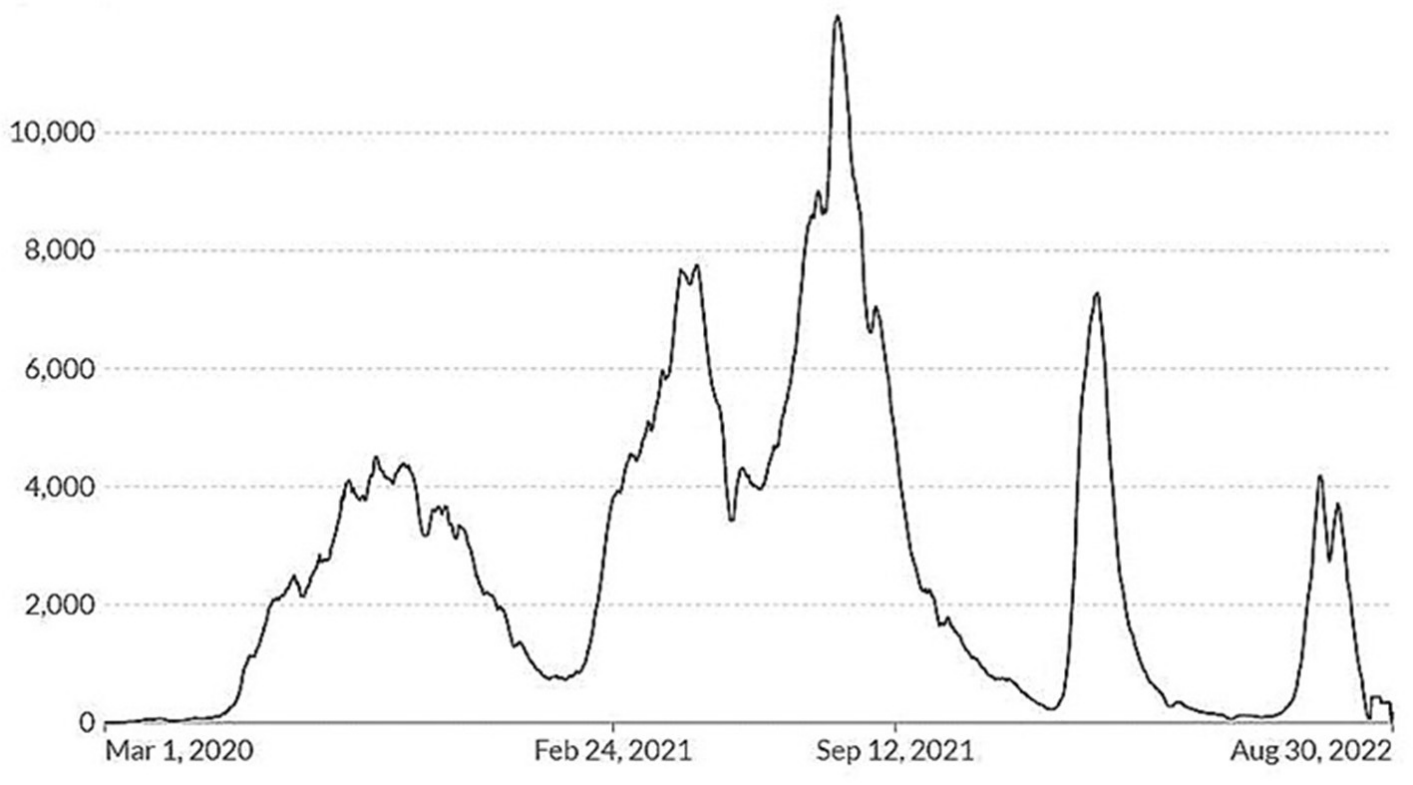
Reported cases of COVID-19 in Iraq (Johns Hopkins University CSSE).

The first case of COVID-19 in Iraq was reported on 24 February 2020 in Najaf, Iraq, by a person who had recently arrived from Iran (1). The first wave peaked in September 2020. A bimodal pattern occupied most, with a nadir occurring in January 2021 and January 2022, followed by peaks in February 2021 and to a lesser extent in February 2022 (2) (Figure 1). This is a similar pattern in Iran but differs somewhat from Syria and Türkiye.

By 27 August 2022, Iraq had reported 2.45 million cases with 25,343 deaths, second to Iran in the number of cases but below Pakistan and Tunisia in the number of deaths in the Eastern Mediterranean region (3). Reported cases are shown in Figure 1. This amounts to 5,798 confirmed cases and 627 deaths per 100,000, with male patients accounting for two-thirds of reported deaths (4). Baghdad, which has 21.3% of Iraq's population, had 28.2 and 21.1% of nationally reported COVID-19 cases and deaths, respectively (5). By the time of this study, Omicron variants were being seen in Iraq (6). Masking, isolation of cases, limiting public exposure, and hospitalization of seriously affected persons in special hospital wards were approaches widely promoted by authorities. Vaccination against COVID-19 began in January 2021. As of August 2022, an estimated 19.4% of the population is fully immunized using one of the three vaccines available: Pfizer-BioNTech, AstraZeneca, or Sinopharm (7). When tracking stopped in March 2023, an estimated 28.2% of Iraqis had been immunized. Widespread distrust of the government, fear of adverse reactions, and lack of knowledge related to the vaccine have been reported (8).

The pandemic found the Iraqi health system seriously unprepared. Sanctions, years of external and internal conflict, and corruption left health services weak. Iraq has chronically under-investing in health, spending 4.9% of Gross Domestic Product (GDP) on health services in 2020 compared with 6.7% by Iran and 7.6% by Jordan (9). When oil prices are depressed, investment in the health sector is even more restricted. Insecurity, workplace violence, and declining living standards have fueled large emigration of healthcare staff, leaving health facilities even more understaffed. COVID-19 has only exacerbated serious underlying health system problems. Special COVID-19 facilities in Iraq have experienced disastrous fires starting in intensive care units, increasing an already high distrust in the government (10).

Reports have examined the implications of the national COVID-19 immunization program and lessons for future outbreaks in Iraq (9). Additional studies have reported the mental health burden of COVID-19 and the impact of the infection on household food security in Iraq (11, 12). There have been efforts to estimate the socioeconomic consequences of the pandemic and population vulnerabilities in Iraq, including a gender analysis (13, 14). The impact of COVID-19 on violence against health workers has also been reported (15, 16). Of the 680 original research articles analyzed covering the mental health impact of COVID-19 retrieved in 2020, there were 40 reported from Iran and 36 from Turkey (17). Most studies have focused on the physical and emotional impact of the pandemic on vulnerable populations. Studies have investigated issues in groups such as health workers, or specific sub-populations. Reports focusing on health workers found a high risk of exposure, overwork, and moral dilemmas, resulting in an increase in mental health problems. There have been limited data reported from cross-sectional studies on the household impact of COVID-19 in urban areas. This study aimed to assess the consequences of COVID-19 infection on households in Baghdad.

This study began in early 2022 to examine a cross-sectional sample of households representatively sampled from Baghdad neighborhoods with the objective of determining specifics of how COVID-19 infections may have affected individual households. Variables examined included household demographics, history of illness, use of COVID-19 precautions, employment, impact on children, and mental health indicators. Although some initial smaller studies have reported some components, our goal was to examine these and other indicators on a random selection of households in Baghdad.

## Methods

### Study design

**Figure 2:**
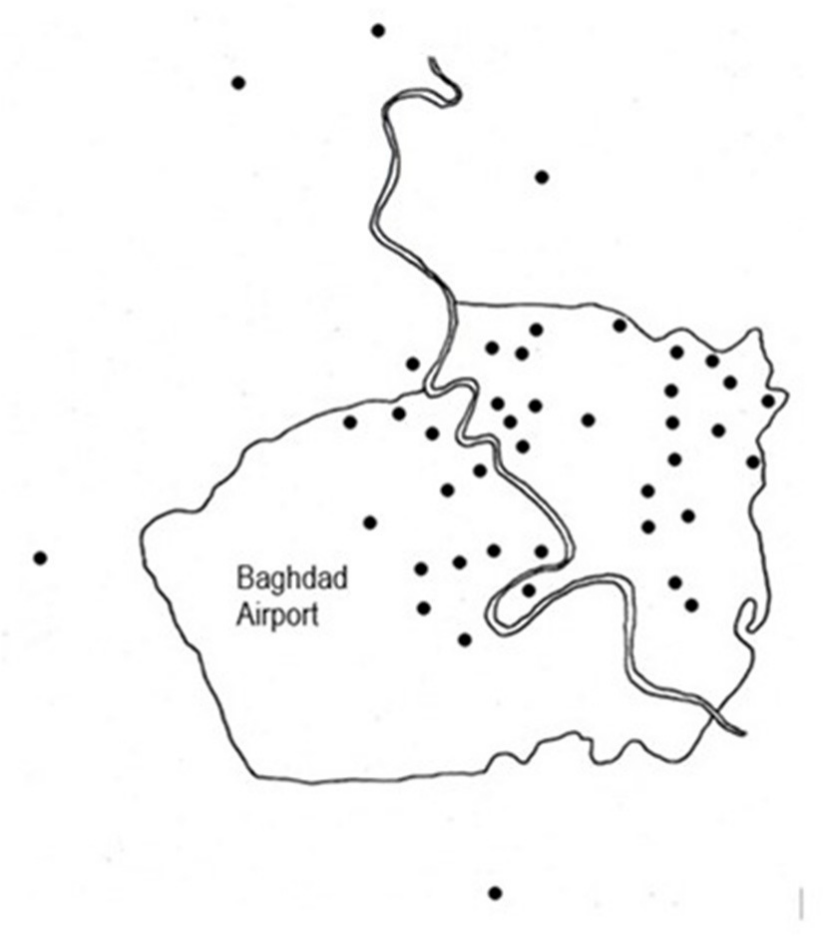
City of Baghdad with location of clusters indicated.

This cross-sectional household survey was conducted from November 2021 to February 2022 in urban and rural parts of Baghdad, with an estimated population of 8.6 million (18). Using a mapping of Baghdad's 22 urban districts, we selected clusters proportional to population size from these urban districts. This process identified 36 clusters which were distributed among 20 of the 22 urban districts. Outside of Baghdad's urban areas, there are five rural sectors. Taking the largest district in each, five additional sites were identified for a total of 41 clusters in the study (Figure 2). For each district, the actual cluster location was identified using geospatial sampling. This allowed the selection of a random residential street and dwelling to start data collection. After interviewing the first household, interviewers moved to the right and continued data collection using a systematic random sampling technique by taking every other dwelling consecutively until 22 households had been interviewed in each cluster. A household was defined as a residential unit with a separate entrance from the street and a unit with a separate kitchen, a standard definition used in Iraqi surveys. Multifamily dwellings were single-story structures and were sampled in the same manner. Multistory multifamily residences did not exist in these areas. Verbal informed consent was obtained from the male head or senior female of the household after the purpose of the interview was explained.

Households who agreed to participate were included.

### Sample size calculation

Setting a CI of 95%, a precision of 0.05, and the anticipated proportion of households having an indicator of interest as 0.5, the estimated sample size required would be 385 households. Using a design effect estimate of 2 to account for clustering gave a sample size of 770. Considering potential refusals to participate and potential security situations, we selected a sample size of 900.

### Data collection

A structured questionnaire was developed, starting with a listing of household members, then seeking information on COVID-19 infections, the main clinical symptoms, receipt of a COVID-19 vaccine, any hospital admissions, and COVID-19 household mortality information. The senior member of the household was also asked about the social and the household economic burden of the pandemic and adherence to the protective measures by household members. The senior member was also asked about their own personal symptoms of anxiety and depressive symptoms.

Survey teams consisted of male and female interviewers (both medical doctors) and a supervisor; all were experienced in household surveys. In some locations, local facilitators familiar with the selected areas assisted teams. A 2-day training on the specifics of this survey was conducted. Following verbal consent of the household head or senior female present, one interviewer asked questions and the other team member recorded responses. Interviews generally took place in the presence of all household members and required about 50 min. No interviews were conducted where the household head or a senior female were not present. The interview written records were checked by interviewers and the supervisor before being translated from Arabic. No personally identifiable information was collected. All data were stored confidentially after collection.

### Analysis

Analysis was conducted in Baghdad and Baltimore using the Stata statistical package (1). Data quality was checked by examining missing responses and inappropriate values. Since missing values accounted for <10%, they were dropped from analyses. All analyses incorporated the complex survey design and sample weights for clustering. The primary unit of analysis was the household member.

Descriptive statistics were calculated to depict sample demographics and estimate the prevalence of COVID-19 vaccination, infection, and related mortality. Descriptive sub-analyses were also conducted to understand symptomatology, hospitalization rates, and mortality among household members reporting previous COVID-19 infection. Pearson's chi-square and Fischer's exact tests were conducted to assess differences in protective measures undertaken during the COVID-19 pandemic, COVID-19's economic and psychosocial impact on the head or senior member of the household by whether the household had exposure to COVID-19 (i.e., report of COVID-19 infection within the household); *p* < 0.05 were considered statistically significant.

## Results

Across 41 survey clusters, 929 households were contacted, of which only two declined to participate (99% participation rate). Among participating households, data on 3949 residents were documented with an average household size of 4.25 persons (range 1–9).

Household demographics are shown in Table 1. The average age of household residents was 29.3 years (28.1–30.3), with 2004 (50.8%) being female. Of those in the workforce, only 1.5% listed their status as unemployed. There were 1,464 (37.1%) household members of the 927 households surveyed who indicated they had been infected by COVID-19. Of these, 1,116 (76.2%) had their infection diagnosed by COVID-19 testing, and the remaining 348 (23.8%) reported a diagnosis based on the presence of characteristic symptoms. Of household members reported to be infected, 73.5% were under the age of 50 years. For the 34 reported COVID-19 deaths, 25 (71.8%) died in hospital, 6 (18.0%) died at home, and 4 (10.3%) of an undisclosed location. Of the 3,138 household residents aged 12 years and above, 1,598 (50.9%) were immunized. Reports of infection with COVID-19 in a household member or a household death from COVID were not any more common in high-density/low-income parts of Baghdad than the areas with lower density.

**Table 1:** Demographic characteristics of household members (*N* = 3,949)^a^.

**Variable name**	***n* (%) or m**	**95% CI**
**Age**		
Age (years)	29.3	28.1, 30.3
**Gender**		
Male	1,945 (49.2)	47.5, 51.0
Female	2,004 (50.8)	49.0, 52.5
**Occupation**		
Unemployed	58 (1.4)	0.9, 2.3
Not in workforce (e.g., children, students, handicapped, retired)	1,858 (47.0)	45.1, 49.0
Domestic labor/housewives	761 (19.3)	17.2, 21.6
Manual labor	111 (2.8)	2.0, 3.9
Clerical work	458 (11.6)	9.3, 14.4
Commerce/trade	327 (8.3)	7.0, 9.7
Food-related work	33 (0.8)	0.5, 1.3
Professionals	252 (6.4)	4.4, 9.2
Military	91 (2.3)	1.5, 3.6
**Ever tested for COVID-19 infection**		
No	2,510 (63.6)	58.7, 68.2
Yes	1,438 (36.4)	31.8, 41.3
Don't know	1 (<0.1)	0.003, 1.7
**Ever infected by COVID-19**		
Uncertain/Unknown	2,484 (62.9)	57.6, 67.9
Likely/probable infection based on symptoms	348 (8.8)	6.7, 11.5
Yes, confirmed with lab test	1,116 (28.3)	23.8, 33.2
**Received COVID-19 vaccination (age 12 and older) at least**		
**1 dose**		
No	1,540 (49.1)	42.1, 56.1
Yes	1,598 (50.9)	43.9, 57.9
**COVID-19 Mortality**		
No	3,915 (99.1)	98.6, 99.5
Yes	34 (0.9)	0.5, 1.4

a Estimates are weighted.

Table 2 shows the common symptoms experienced by the 1,464 persons with a diagnosis of COVID-19. All reported symptoms were significantly (*p* < 0.001) more common in the 117 patients who were hospitalized than among those treated at home, with the exception of headache and sore throat. In 83 (80.3%; 95%CI: 66.8, 89.3), difficulty breathing was the symptom most commonly leading to hospitalization. The average length of hospital stay was 16.7 days (95%CI: 12.1, 21.3). In those dying from COVID-19, the average number of days between onset of symptoms and death was 26 days (95%CI: 13.8, 37.2), data not shown.

**Table 2:** Symptoms and age profile of household members diagnosed with COVID-19 (*N* = 1464)^a, b^.

**Symptoms**	***n* (%)**	**95% CI**
Fever	1,011 (69.0)	65.0, 72.8
Fatigue	920 (62.9)	58.0, 67.5
Loss of appetite	856 (58.5)	53.9, 62.9
Loss of taste	771 (53.1)	46.2, 59.8
Loss of smell	666 (45.5)	39.8, 51.3
Chills	641 (43.8)	38.8, 48.8
Cough	619 (42.3)	33.2, 51.9
Muscle/body pains	576 (39.3)	32.3, 46.8
Dyspnea	571 (39.0)	30.8, 47.8
GIT symptoms	431 (29.4)	24.6, 34.7
Headache	25 (1.7)	0.8, 3.9
Sore throat	5 (0.4)	0.1, 1.1
Other	49 (3.4)	1.8, 6.2
**Age profile**		
Age	Number	Percent
<15	129	8.8%
15–19	90	6.1%
20–29	280	19.2%
30–39	329	22.5%
40–49	248	16.9%
50+	388	26.5%

a Estimates are weighted.

b Diagnosis was by test or symptomatology.

Table 3 sets out the various protective measures that household heads reported taking to limit infection. In some households, more precautions were taken if there had previously been someone ill with COVID-19. Households that reported a previous COVID-19 infection were somewhat less likely to use protective measures than those without a reported case, though this trend was not statistically significant. When the vaccination status of the head of household is considered, those immunized reported they were significantly more likely to have mostly or always adhered to protected measures such as masking, limited social contacts, and handwashing during the pandemic, compared to those unvaccinated (OR: 2.98 95%CI: 1.38, 6.40).

**Table 3:** Use of protective measures among heads of households according to reported COVID-19 infections (*N* = 929)^a^.

	**Total (*N =* 929)**		**Individuals with no COVID cases w/in household (*N =* 322)**		**Individuals with COVID cases w/in household (*N =* 607)**		***p*-value**
	***n*** **(%)**	**95% CI**	***n*** **(%)**	**95%CI**	***n*** **(%)**	**95%CI**	
**Avoid crowding and social contact**							
No	120 (13.0)	9.2, 18.0	33 (10.1)	6.4, 15.7	88 (14.5)	9.6, 21.2	0.137
Yes	651 (70.1)	63.4, 76.0	244 (76.0)	69.3, 81.5	407 (67.0)	58.3, 74.7	
Sometimes	157 (16.9)	12.7, 22.2	45 (13.9)	8.9, 21.2	112 (18.5)	13.4, 25.0	
**Limit social contact during the holidays**							
No	307 (33.1)	25.4, 71.8	94 (29.2)	20.5, 39.9	213 (35.1)	26.7, 44.5	0.194
Yes	622 (66.9)	58.2, 74.7	228 (70.8)	60.1, 79.5	394 (64.9)	55.5, 73.3	
**Wash hands more frequently than before the pandemic**							
No	72 (7.8)	5.1, 11.6	25 (7.9)	5.1, 12.1	47 (7.7)	4.6, 12.6	0.076
Only after being in public	128 (13.7)	8.7, 21.0	30 (9.3)	5.4, 15.6	98 (16.0)	9.8, 26.3	
Yes	729 (78.4)	70.1, 85.0	266 (82.5)	75.8, 87.7	463 (76.3)	65.5, 84.5	
Other	1 (0.1)	0.01, 0.7	1 (0.3)	0.04, 2.1	0	0	
**Wear a mask when outside the house**							
No	120 (12.9)	9.5, 17.3	32 (9.9)	6.3, 15.1	88 (14.5)	10.4, 19.9	0.168
Yes	587 (63.4)	55.9, 70.2	217 (67.7)	59.1, 75.2	370 (61.1)	52.5, 69.1	
Sometimes	220 (23.7)	19.0, 29.2	72 (22.5)	15.9, 30.7	148 (24.4)	19.4, 30.1	
**Follow curfew**							
No	155 (16.8)	12.4, 22.3	51 (15.9)	10.3, 23.7	104 (17.3)	12.3, 23.6	0.709
Yes	768 (83.2)	77.7, 87.6	270 (84.1)	76.3, 89.7	498 (82.7)	76.4, 87.7	

a Estimates are weighted.

In Table 4, information on the economic impact of COVID-19 on households is set out. Excepting the costs related to laboratory testing, medications, and hospitalization, as well as the impact of having to stop working to support an ill family member, there was no significant direct household economic impact of a household case of COVID-19 reported, except for loss of income during lockdowns.

**Table 4:** Economic impact of COVID-19^a^.

	**Total (*N =* 904)**		**Individuals with no COVID cases w/in household**		**Individuals with COVID cases w/in household**		**p-value**
	***n*** **(%)**	**95% CI**	***n*** **(%)**	**95%CI**	***n*** **(%)**	**95%CI**	
**Incurred expenses due to laboratory testing and medicines (*****N** =* **904)**							
No	370 (41.0)	34.2, 48.1	213 (66.9)	58.6, 74.2	157 (26.9)	19.5, 35.9	<0.001
Yes	534 (59.0)	51.9, 65.8	106 (33.2)	25.8, 41.4	428 (73.1)	64.2, 80.5	
**Incurred expenses due to hospitalization (*****N** =* **905)**							
No	777 (86.3)	80.5, 90.6	301 (94.5)	85.6, 98.0	477 (81.9)	75.9, 86.7	0.003
Yes	123 (13.7)	9.4, 19.5	18 (5.5)	2.0, 14.5	105 (18.1)	13.4, 24.1	
**Voluntarily stopped working (*****N** =* **900)**							
No	591 (65.2)	58.5, 71.4	230 (73.2)	64.8, 80.2	360 (61.0)	53.3, 68.2	0.009
Yes	314 (34.8)	28.6, 41.5	84 (26.8)	19.8, 35.2	230 (39.0)	31.8, 46.7	
**Stopped working to care for family member (*****N** =* **908)**							
No	722 (79.5)	71.8, 85.5	295 (92.8)	87.2, 96.1	427 (72.3)	62.9, 80.1	<0.001
Yes	186 (20.5)	14.5, 28.2	23 (7.2)	3.9, 12.8	164 (27.7)	19.9, 37.1	
**Lost job (*****N** =* **908)**							
No	713 (78.5)	73.5, 82.8	248 (77.9)	69.5, 84.5	465 (78.8)	73.9, 83.1	0.206
Yes, due to pandemic	82 (9.0)	5.9, 13.4	21 (6.6)	3.5, 12.2	61 (10.3)	6.7, 15.5	
Yes, other reason	46 (5.0)	3.5, 7.11	19 (6.1)	3.5, 10.3	26 (4.5)	3.0, 6.6	
Yes, unknown reason	67 (7.5)	4.7, 11.7	30 (9.4)	5.1, 16.8	38 (6.4)	3.9, 10.4	
**Lost job due to pandemic and still unemployed at time of survey (*****N*** = **82)**							
No	42 (51.6)	38.2, 64.8	11 (50)	30.3, 69.8	32 (52.2)	34.9, 68.9	0.879
Yes	40 (48.4)	35.2, 61.8	11 (50)	30.3, 69.8	29 (47.8)	31.1, 65.1	
**Reduction in income due to lockdowns (*****N** =* **900)**							
No	556 (61.7)	55.1, 68.0	214 (68.5)	59.6, 76.2	342 (58.2)	50.6, 65.4	0.037
Yes	344 (38.3)	32.1, 44.9	98 (31.5)	23.8, 40.4	246 (41.8)	34.6, 49.4	
**Difficulty paying rent/household expenditures (*****N** =* **910)**							
No	290 (31.9)	24.6, 40.2	94 (29.6)	20.3, 40.9	196 (33.1)	23.4, 41.9	0.225
Yes	176 (19.3)	14.3, 25.7	51 (16.0)	10.3, 24.1	125 (21.1)	15.2, 28.5	
N/A	444 (48.8)	38.0, 59.7	173 (54.4)	40.5, 67.7	271 (45.8)	35.0, 57.0	
**Not enough money to purchase food (*****N** =* **905)**							
No	724 (80.0)	74.1, 84.9	252 (80.2)	71.1, 86.9	472 (79.9)	73.6, 85.1	0.948
Yes	181 (20.0)	15.1, 25.9	62 (19.8)	13.1, 28.9	119 (20.1)	14.9, 26.5	
**Not enough money to purchase usual medicines for family members (*****N** =* **903)**							
No	674 (74.7)	67.7, 80.6	250 (79.3)	69.9, 86.4	424 (72.2)	63.6, 79.4	0.175
Yes	229 (25.3)	19.4, 32.3	65 (20.7)	13.6, 30.1	164 (27.8)	20.6, 36.4	
**Not enough money to pay for utilities (electricity or gas) (*****N** =* **900)**							
No	743 (82.5)	76.4, 87.4	261 (83.0)	74.4, 89.1	482 (82.3)	74.9, 87.8	0.870
Yes	157 (17.5)	12.7, 23.7	54 (17.0)	10.9, 25.7	103 (17.7)	12.2, 25.1	
**Financially supported family members due to financial problems caused by pandemic (*****N** =* **899)**							
No	404 (45.0)	38.0, 52.2	135 (42.8)	34.3, 51.7	269 (46.2)	38.3, 54.2	0.458
Yes	495 (55.0)	47.8, 62.0	181 (57.2)	48.3, 65.7	314 (53.8)	45.8,61.7	

a Estimates are weighted.

The data in Table 5 showed that most school children were out of school because of school closure. Of children who left school during the COVID-19 pandemic, 71.8% of households reported that family members took on teaching roles at home for the children.

**Table 5:** Impact of COVID-19 on households with children (*N* = 490).

	***n*(%)**	**95%CI**
**My children left school due to pandemic**		
No	30 (6.1)	3.2, 11.3
Yes	460 (93.9)	88.7, 96.8
**Reasons why children left school (*****N** =* **460)**		
School was closed	456 (99.1)	97.2, 99.7
Parents were afraid of infection	39 (8.5)	4.7, 14.7
Economic reasons	16 (3.5)	1.4, 8.2
**Parents/family members in the household took on a teaching role for students who left school during pandemic (*****N** =* **460)**		
No	130 (28.2%)	20.9, 37.0
Yes	330 (71.8%)	63.0, 79.1

In Table 6, the differences are set out for emotional symptoms reported by the head of household or senior household member between those in households where a COVID-19 case had been present and those where there were no COVID-19 cases reported. Most of the anxiety and depressive symptoms were reported significantly more commonly from those in households that had experienced a case of COVID-19. The non-significant exceptions were suicidal ideation, trouble concentrating, alternations in appetite, difficulties in sleeping, feeling lonely, and the need to take medications to control anxiety.

**Table 6:** Anxiety and depression symptoms reported by the senior member of the household^a^.

**Anxiety symptoms: Over the last 2 weeks, frequency of the following:**				**Depression symptoms: Over the last 2 weeks, frequency of the following:**			
	**Total (*****N** =* **914)**	**Individuals with no COVID cases w/in household (*****N** =* **319)**	**Individuals with COVID cases w/in household (*****N** =* **395)**		**Total (*****N** =* **914)**	**Individuals with no COVID cases w/in household (*****N** =* **319)**	**Individuals with COVID cases w/in household (*****N** =* **395)**
	***n*** **(%) 95%CI**	***n*** **(%) 95%CI**	***n*** **(%) 95%CI**		***n*** **(%) 95%CI**	***n*** **(%) 95%CI**	***n*** **(%) 95%CI**
**Nervous, anxious, or on edge** ^*^				**Little interest or pleasure in doing things** ^*^			
No	450 (49.2) 38.3, 60.2	197 (61.7) 49.3, 72.8	253 (42.5) 31.7, 54.9	No	577 (63.2) 52.5, 72.7	240 (75.2) 63.3, 84.2	337 (56.7) 43.8, 68.8
Yes	464 (50.8) 39.8, 61.7	122 (38.3) 27.3, 50.7	342 (57.5) 45.1, 68.9	Yes	337 (36.8) 27.3, 47.5	79 (24.8) 15.8, 36.7	258 (43.3) 31.2, 56.2
**Not being able to stop or control worrying** ^*^				**Down, depressed, or hopeless** ^*^			
No	492 (53.9) 42.8, 64.8	205 (64.2) 52.4, 74.5	287 (48.3) 36.4, 60.4	No	635 (69.4) 59.4, 77.9	252 (79.1) 67.0, 87.6	382 (64.3) 52.0, 74.9
Yes	422 (46.2) 35.4, 57.3	114 (35.8) 25.5, 47.7	308 (51.7) 39.6, 63.6	Yes	279 (30.6) 22.1, 40.6	67 (20.9) 12.5, 33.0	213 (35.7) 25.1, 48.0
**Worrying too much about different things** ^*^				**Feeling lonely** ***ns***			
No	504 (55.2) 43.7, 66.2	216 (67.8) 55.9, 77.7	288 (48.5) 36.0,61.1	No	668 (73.1) 62.4, 81.6	261 (81.8) 70.6, 89.4	407 (68.4) 54.8, 79.4
Yes	410 (44.8) 33.8, 56.3	103 (32.2) 22.3, 44.1	307 (51.6) 38.9, 64.0	Yes	246 (26.9) 18.4, 37.6	58 (18.2) 10.6, 29.4	188 (31.6) 20.6, 45.2
**Trouble relaxing** ^*^				**Trouble falling or staying asleep, or sleeping too much** ***ns***			
No	556 (60.9) 49.7, 71.0	234 (73.6) 62.2, 82.5	322 (54.1) 40.9, 66.7	No	607 (66.4) 53.2, 77.5	233 (73.2) 60.4, 83.1	374 (62.8) 47.8, 75.7
Yes	358 (39.1) 29.0, 50.3	84 (26.4) 17.5, 37.8	274 (45.9) 33.3, 59.1	Yes	307 (33.6) 22.5, 46.8	85 (26.8) 16.9, 69.7	222 (37.2) 24.3, 52.2
**Being so restless that it is hard to sit still** ^*^ ^b^				**Tired or having little energy** ^*^ ^b^			
No	623 (68.2) 57.9, 73.1	256 (80.4) 69.3, 88.2	367 (61.7) 48.9, 73.1	No	554 (60.7) 47.9, 72.2	226 (71.0) 57.8, 81.4	328 (55.2) 41.2, 68.4
Yes	290 (31.8) 23.0, 42.1	62 (19.6) 11.9, 30.7	228 (38.3) 26.9, 51.1	Yes	359 (38.3) 27.8, 52.1	92 (29.0) 18.6, 42.2	267 (44.8) 31.6, 58.8
**Easily annoyed or irritable** ^*^				**Poor appetite or overeating** ***ns***			
No	528 (57.8) 47.7, 67.3	224 (70.2) 60.0, 78.8	304 (51.1) 39.0, 63.1	No	614 (67.2) 53.4, 78.5	238 (74.7) 61.0, 84.7	376 (63.2) 47.7, 76.3
Yes	386 (42.2) 32.7, 61.0	95 (29.8) 21.3, 39.9	291 (48.9) 36.9, 61.0	Yes	300 (32.8) 21.5, 46.6	81 (25.3) 15.3, 39.0	219 (36.8) 23.7, 52.3
**Nervous, anxious, or on edge** ^*^				**Little interest or pleasure in doing things** ^*^			
**Afraid that something awful might happen** ^*^ ^b^				**Trouble concentrating on things**^c^ ***ns***			
No	529 (57.9) 46.3, 68.8	219 (68.6) 56.4, 78.6	310 (52.2) 39.6, 64.6	No	631 (69.6) 57.5, 79.6	246 (77.1) 65.6, 85.7	385 (65.6) 50.9, 77.8
Yes	384 (42.1) 31.2, 53.7	100 (31.4) 21.4, 43.6	284 (47.8) 35.4, 60.5	Yes	275 (30.4) 20.4, 42.5	73 (22.9) 14.4, 34.4	202 (34.4) 22.2, 49.1
**Need to take medicines to control emotions** ***ns***				**Suicidal ideation (thoughts better off dead or hurting self)**^b^ ***ns***			
No	758 (83.0) 75.8, 86.8	281 (88.2) 79.1, 93.6	477 (80.2) 71.4, 86.8	No	795 (87.1) 80.3, 91.8	280 (87.9) 77.4, 93.8	515 (86.7) 78.5, 92.1
Yes	156 (17.0) 11.6, 24.2	38 (11.8) 6.4, 20.10	118 (19.8) 13.2, 28.6	Yes	118 (12.9) 8.2, 19.7	39 (12.2) 6.2, 22.6	79 (13.3) 7.9, 21.5

a Estimates are weighted;

b 1 missing;

c 8 missing.

* p < 0.05; ^**^p < 0.001 ns not significant.

## Discussion

This represents the only cross-sectional survey to look at the household impact of COVID-19 in Baghdad. Indeed, few studies have attempted a cross-sectional examination of the COVID-19 impact on major cities in low- and middle-income countries. In these sample data from 3,949 household members from 927 randomly selected households in Baghdad at the time of the study, there were 1,464 persons (37.1%) who had experienced COVID-19 confirmed either by testing of nasopharyngeal swabs (1116) or by characteristic symptoms (348). Among the survey population, there were 34 deaths attributed to COVID-19. Of the eligible study population in Baghdad, 50.9% had received one or more COVID-19 immunizations. Iraq as a whole had received at the time of this study sufficient vaccine to fully immunize 27.8% of its total population, suggesting that Baghdad was more fully immunized than other parts of Iraq (18, 19).

Some of the clusters for this survey were in high-density/lower-income neighborhoods. COVID-19 cases were not reported any more frequently from these areas. We had anticipated potentially higher infection frequencies in these areas, as suggested by other studies in high-density population areas (20). Analysis of data from South Korea indicates that social connectivity is also an important predictor of infection, something not assessed in our study (21). In our study, the household size (4.75 persons) was considerably less than in our earlier studies, which found household size to be 5.7 (22). The implications of these smaller sized households on susceptibility to infection and transmission in Baghdad are uncertain. Data from New York City suggested that larger household size was the most important driver in the variation of COVID-19 incidence rates (23).

### Household COVID-19 precaution

Of interest were the precautions that households took to avoid COVID-19 infection. At least one dose of COVID-19 vaccine was reported to have been received by half of household members (50.9%) over the age of 12 years. The majority of households reported social distancing, increased hand washing, observing curfews, and wearing masks. The frequency with which these steps were reported was broadly similar to that reported earlier in other countries, such as Malaysia (24). Other studies have found considerable variation in compliance with preventive recommendations by sex and socioeconomic status (25, 26). We found no statistical differences in taking these precautions among households where there had been a case of COVID-19 reported compared with those not reporting a case, although the trend was for less observance of precautions in households where COVID-19 cases had occurred. Although not conclusive, this could suggest that having experienced a household case of COVID-19 increased a sense of coping capacities and lessened the fear of infection, causing the household to reduce its use of precautions. A similar phenomenon was noted among 344 persons in the UK (27). The vaccination of the head of household did significantly predict increased compliance with preventive measures by household members.

### Financial impact

Not surprisingly, the reported presence of a case of COVID-19 in the household resulted in increased costs when compared with households not reporting cases. These additional costs from affected households were for testing, treatment, and hospitalizations. A financial impact was also reported in those households where a household member stopped employment to care for a person ill with COVID-19. There were no other direct economic outcomes that differed significantly between households that experienced COVID-19 and those that had not.

However, overall, many households reported a financial impact that they attributed to the pandemic, unrelated to a history of an infected person in the household. One-third of households reported that someone voluntarily stopped working because of the pandemic. A further one-third of households reported financial consequences arising from the lockdown. There were 10.3% of households that reported a household member having lost employment because of the pandemic, with half still unemployed at the time of the survey. In Nigeria, a rise in costs related to COVID-19 precautions was noted, but in Nigeria, the costs of other household expenses were reduced during lockdown periods (28). While the Baghdad data showed no significant differences in the pandemic economic impact between high-density low-income housing areas and low-density areas, a Kampala report indicated that slum dwellers experienced major consequences to household income and food supplies. In addition, the Uganda study noted an increase in domestic violence, child labor, and teenage pregnancies, events not recorded in our study (29).

### School-age children

In households with school-age children, only 3.7% were in school during the pandemic. The impact on the learning of children is certainly immense and will be felt for some time (30). This will be particularly true for children already in adverse situations, either at home or in the larger environment. Although Iraqi schools opened for 1–2 days a week earlier in the pandemic than some countries, the impact of closure was considerable as Iraq has one of the youngest populations in the region (31). Although some e-learning sites were available, these had limited accessibility in places. Nearly three-quarters of households reported taking on home teaching responsibilities during this time.

### Emotional impact

Of concern was the emotional impact of the outbreak reported by Baghdad households. For almost every category of anxiety and depressive symptoms, the senior household member interviewed in households where there had not been a case of COVID-19 reported significantly fewer symptoms than were reported from a household where a disease case had been reported. This impact was similar to the findings of an Iran study (32). However, the overall levels of anxiety and depression seem high in this Baghdad population, even for households not directly affected by COVID-19. While worldwide, there were increases in anxiety and depression associated with the epidemic, the continuing civil instability, the high-profile fires in Baghdad's COVID-19 hospital wards, and reported assaults on health workers probably amply this stress (33, 34). Worldwide, there has been considerable attention given to mental health and COVID-19. In Arab countries, the information has been less forthcoming. Data from seven Arab countries found that COVID-19-related stress was highest in Egypt, followed by Palestine and Iraq, but with considerable variations among countries (35). Work by Lafta and Mawlood (11) found depression and anxiety were increased among persons attending medical facilities for symptoms of COVID-19 (17). Much of this stemmed from concern with personal and family health as well as social and economic burden attributed to COVID-19. Similar concerns were seen in Kuwait and Turkey (36, 37).

### Limitations

A study of this nature has a variety of limitations. Although all efforts were made to secure a representative random sample of Baghdad households, this, in fact, might not have occurred. This could be a particular concern in some of the more peripheral areas of Baghdad, which may not have been fully included in the sampling process. Infection rates were similar across Baghdad, though a large sample size study may have found differences among high- and low-density areas of Baghdad. Senior household members may not have adequately reported the impact of COVID-19 on the household, with diminished recall about earliest events. There may have been some confusion about the diagnosis of COVID-19, where there was reliance only on symptoms for the classification in 23.8%. Respondents were not able to report if a positive COVID-19 test in a household member was the result of a PCR or antigen test, so some test result uncertainty was present. Death certificates were not examined, so the deaths reported by households due to COVID-19 were not verified, and the role of co-morbidities was not evaluated. We accepted reports of COVID-19 immunizations in household members but did not check actual records. The qualitative reports were recorded as stated, with limited opportunity for in-depth interviews. This was particularly true of the mental health symptoms, as a cross-sectional offered little opportunity to probe the description and nature of the symptoms.

### Interpretation

COVID-19 had a substantial impact on households in Baghdad. There were financial issues in a substantial percentage of households, though few could be directly linked to the presence of COVID-19 infections in the specific household. In some cases, households having experienced cases reported a greater financial impact, but in most cases, the differences with households without infection were not statistically significant. Observance of precautions was widely reported, including half of persons aged 12 years and older having received at least one vaccine dose. Of great concern was the level of emotional stress reported. While some are certainly related to the pandemic, there are many other stressors occurring in society concurrently. The capacity of the health services to provide mental health services for this population is extremely limited. In the current unstable environment of Iraq, it is unlikely that this high level of emotional stress will dissipate easily.

Although contexts may differ among countries, the pattern seen in Iraq is similar to some other countries in the region, although some, such as Iran, had a greater burden of disease. The inclusion of mental health in a surveillance system during a pandemic could provide early warning of population needs. Should a return of COVID-19 reach serious levels, these observations on household precautions and potential impact of household infections would help identify appropriate communications strategies and interventions. Better understanding the mental health consequences of a pandemic could help health resources be better able to provide resources. On a positive note, the reported use of household protective measures in Baghdad is evidence of success in communication strategies. While some households, directly or indirectly affected by COVID-19, did have financial constraints, the need for external financial assistance was less than some had anticipated.

## Data availability statement

The raw data supporting the conclusions of this article will be made available by the authors, without undue reservation.

## Ethics statement

The studies involving humans were approved by the Scientific Committee of the Department of Community Medicine, Al Mustansiriya University (approval no. 5092). The JHSPH Institutional Review Board declared analysis of de-identified data as exempt non-human subjects' research (decision no. 22327). The studies were conducted in accordance with the local legislation and institutional requirements. The Ethics Committee/institutional review board waived the requirement of written informed consent for participation from the participants or the participants' legal guardians/next of kin because Verbal consent was obtained, as written consent is often seen by Iraqis as a potential breech of confidentiality, and illiterate persons are hesitant to agree to a document they cannot read.

## Author contributions

RL and GB conceived of the study and created the study design. SA-S assisted with the implementation plan, managed data collection, and initial analysis. MM conducted a detailed analysis. GB led in writing the manuscript, assisted by RL and MM. All authors have reviewed and approved the submitted manuscript and take full responsibility for the contents.

## References

[1] WHO. COVID-19 Summary of Annual Report. Iraq. (2020). Available online at: https://C:/Users/LocalAdmin/Dropbox/PC/Downloads/Iraq_WHO_COVID19_summary%20report_2020_Final.pdf (accessed August 25, 2022).

[2] Johns Hopkins University, CSSE. (2020). Available online at: https://coronavirus.jhu.edu/region/iraq (accessed August 19, 2022).

[3] Johns Hopkins University, CSSE. (2020). Available online at: https://coronavirus.jhu.edu/region/ (accessed August 19, 2022).

[4] Mawlood NA Lafta R. Trends in COVID-19, Incidence, mortality, and case fatality in Iraq. Saudi Med J. (2022) 43:500–7. 10.15537/smj.2022.43.5.20220088 35537730 PMC9280596

[5] Reuters COVID-19 Tracker. (2020). Available online at: https://www.reuters.com/world-coronavirus-tracker-and-maps/graphics/world-coronavirus-tracker-and-maps/countries-and-territories/iraq/ (accessed August 19, 2022).

[6] Majed SO Mustafa SA Jalal PJ Fatah MH Miasko M Jawhar Z. SARS-CoV-2 omicron variant genomic and phylogenetic analysis in Iraqi Kurdistan region. Genes. (2023) 14:173. 10.3390/genes14010173 36672914 PMC9859166

[7] Johns Hopkins University. (2020). Available online at: https://coronavirus.jhu.edu/region/iraq (accessed August 30, 2022).

[8] World Bank Group. COVID-19 Vaccine Inequities and Hesitancy in Iraq. (2020). Available online at: https://www.worldbank.org/en/country/iraq/publication/covid-19-vaccine-inequities-and-hesitancy-in-iraq (accessed August 19, 2022).

[9] Lami F Rashak HA Khaleel HA Mahdi SG Adnan F Khader YS. Iraq experience in handling the COVID-19 pandemic: implications of public health challenges and lessons learned for future epidemic preparedness planning. J Public Health. (2021) 43:iii19–28. 10.1093/pubmed/fdab369 34651194 PMC8660009

[10] BBC. Iraq Hospital Fire: Protests as COVID Ward Blaze Kills at Least 92. (2020). Available online at: https://www.bbc.com/news/world-middle-east-57814954 (accessed August 19, 2022).

[11] Lafta RK Mawlood NA. Mental and social burden of COVID-19 on the Iraqi people. Int J Soc Psychiatry. (2023) 69:2007. 10.1177/00207640221077618 35176881 PMC9939619

[12] WFP, World Bank, IFAD, FAO. Food Security in Iraq Impact of COVID-19. (2020). Available online at: https://iraq.un.org/en/149804-food-security-iraq-impact-covid-19-special-section-water-shortages-and-adaptation-november (accessed August 25, 2022).

[13] OXFAM. Gender Analysis of the COVID-19 Pandemic in Iraq. (2020). Available online at: https://policy-practice.oxfam.org/resources/gender-analysis-of-the-covid-19-pandemic-in-iraq-conducted-in-kirkuk-diyala-and-621007/#:~:text=in%20different%20ways (accessed August 19, 2022).

[14] UNICEF/World Bank. Assessment of COVID-19 Impact on Poverty and Vulnerability in Iraq, July 2020. (2020). Available online at: https://www.unicef.org/iraq/media/1181/file/Assessment_of_COVID-19_Impact_on_Poverty_and_Vulnerability_in_Iraq.pdf (accessed August 19, 2022).

[15] Lafta R Qusay N Mary M Burnham G. Violence against doctors in Iraq during the time of COVID-19. PLoS ONE. (2021) 16:e0254401. 10.1371/journal.pone.0254401 34358239 PMC8345879

[16] Ghaleb Y Lami F Al Nsour M Rashak HA Samy S Khader YS. Mental health impacts of COVID-19 on healthcare workers in the Eastern Mediterranean Region: a multi-country study. J Public Health. (2021) 43:34–42. 10.1093/pubmed/fdab321 34642765 PMC8524602

[17] Chen Y Zhang X Chen S Zhang Y Wang Y Lu Q. Bibliometric analysis of mental health during the COVID-19 pandemic. Asian J Psychiatr. (2021) 65:102846. 10.1016/j.ajp.2021.102846 34562753 PMC8435062

[18] WHO, Iraq. Iraq Annual Report 2021. Available online at: https://applications.emro.who.int/docs/9789290210191-eng.pdf?ua=1 (accessed September 22, 2023).

[19] Johns Hopkins University Coronavirus Resources Center. (2020). Available online at: https://coronavirus.jhu.edu/region/iraq (accessed October 3, 2022).

[20] Rader B Scarpino SV Nande A Hill AL Adlam B Reiner RC. Crowding and the shape of COVID-19 epidemics. Nat Med. (2020) 26:1829–34. 10.1038/s41591-020-1104-0 33020651

[21] Jo Y Hong A Sung H. Density or connectivity: what are the main causes of the spatial proliferation of COVID-19 in Korea? Int J Environ Res Public Health. (2021) 18:5084. 10.3390/ijerph18105084 34065031 PMC8150374

[22] Jensen GW Lafta R Burnham G Hagopian A Simon N Flaxman AD. Conflict-related intentional injuries in Baghdad, Iraq, 2003-2014: a modeling study and proposed method for calculating burden of injury in conflict. PLoS Med. (2021) 18:e1003673. 10.1371/journal.pmed.1003673 34351908 PMC8376016

[23] Federgruen A Naha S. Crowding dominate demographic attributes in COVID-19 cases. Int J Infect Dis. (2021) 102:509–16. 10.1016/j.ijid.2020.10.063 33217575 PMC7833246

[24] Azlan AA Hamzah MR Sern TJ Ayub SH Mohamad E. Public knowledge, attitudes and practices towards COVID-19: a cross-sectional study in Malaysia. PLoS ONE. (2020) 15:e0233668. 10.1371/journal.pone.0233668 32437434 PMC7241824

[25] Lee GB Jung SJ Yiyi Y Yang JW Thang HM Kim HC. Socioeconomic inequality in compliance with precautions and health behavior changes during the COVID-19 outbreak: an analysis of the Korean community health survey 2020. Epidemiol Health. (2022) 44:e2022013. 10.4178/epih.e2022013 35008144 PMC8989472

[26] Cabot J Bushnik T. Compliance with precautions to reduce the spread of COVID-19 in Canada. Health Rep. (2022) 33:3–10. 36153709 10.25318/82-003-x202200900001-eng

[27] Harper CA Satchell LP Fido D Latzman RD. Functional Fear Predicts Public Health Compliance in the COVID-19 Pandemic. Int J Ment Health Addict. (2021) 19:1875–88. 10.1007/s11469-020-00281-5 32346359 PMC7185265

[28] Fatoye F Gebrye T Arije O Fatoye CT Onigbinde O Mbada CE. Economic Impact of COVID-19 lockdown on households. Pan Afr Med J. (2021) 40:225. 10.11604/pamj.2021.40.225.27446 35145587 PMC8797048

[29] Nuwematsiko R Nabiryo M Bomboka JB Nalinya S Musoke D Okello D. Unintended socio-economic and health consequences of COVID-19 among slum dwellers in Kampala, Uganda. BMC Public Health. (2022) 22:88. 10.1186/s12889-021-12453-6 35027023 PMC8757926

[30] Sultana F Bari R Munir S. Impact of school closures due to COVID-19 on education in low- and middle-income countries. J Glob Health Rep. (2022) 6:e2022034. 10.29392/001c.36902

[31] Al Jazzera. COVID-19: 10 Million Iraqi Children Back to School. (2022). Available online at: https://www.aljazeera.com/news/2020/11/29/covid-19-10-million-iraqi-children-back-to-school (accessed August 25, 2022).

[32] Moghanibashi-Mansourieh A. Assessing the anxiety level of Iranian general population during COVID-19 outbreak. Asian J Psychiatr. (2020) 51:102076. 10.1016/j.ajp.2020.102076 32334409 PMC7165107

[33] COVID-19 Mental Disorders Collaborators. Global prevalence and burden of depressive and anxiety disorders in 204 countries and territories in 2020 due to the COVID-19 pandemic. Lancet. (2021) 398:1700–1712. 10.1016/S0140-6736(21)02143-7 34634250 PMC8500697

[34] Al Jereera. Dozens Dead as Fire Rips Through Iraq COVID Ward. (2022). Available online at: https://www.aljazeera.com/news/2021/7/12/at-least-20-killed-in-iraq-covid-19-ward-fire-health-official (accessed August 30, 2022).

[35] Shuwiekh HAM Kira IA Sous MSF Ashby JS Alhuwailah A Baali SBA. The differential mental health impact of COVID-19 in Arab countries. Curr Psychol. (2022) 41:5678–92. 10.1007/s12144-020-01148-7 33162726 PMC7605480

[36] Burhamah W AlKhayyat A Oroszlányová M AlKenane A Almansouri A Behbehani M. The psychological burden of the COVID-19 pandemic and associated lockdown measures: experience from 4000 participants. J Affect Disord. (2020) 277:977–85. 10.1016/j.jad.2020.09.014 33065842 PMC7476447

[37] Ozdin S Bayrak Ozdin S. Levels and predictors of anxiety, depression and health anxiety during COVID-19 pandemic in Turkish society: the importance of gender. Int J Soc Psychiatry. (2020) 66:504–11. 10.1177/0020764020927051 32380879 PMC7405629

